# Mapping Changes in Inequities in COVID-19 Vaccinations Relative to Deaths in Chicago, Illinois

**DOI:** 10.5888/pcd20.220319

**Published:** 2023-04-27

**Authors:** Brian Phillips, Lawrence Baker, Laura J. Faherty, Jeanne S. Ringel, Ashley M. Kranz

**Affiliations:** 1 RAND Corporation, Santa Monica, California; 2 Pardee RAND Graduate School, Santa Monica, California; 3 Maine Medical Center, Portland, Maine

**Figure:**
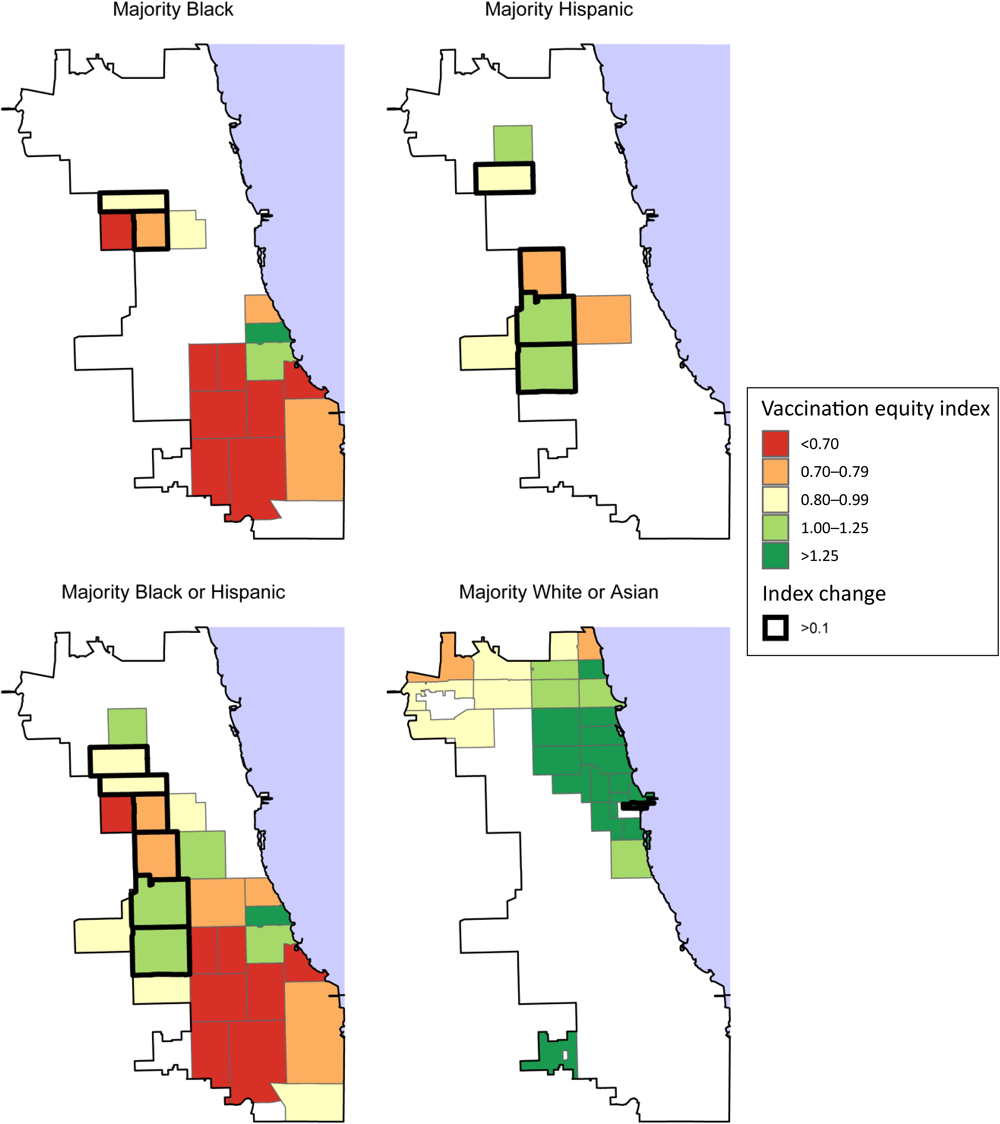
COVID-19 vaccination equity index by zip code, Chicago, Illinois, July 2022, and improvements in equity index since June 2021. The maps display the COVID-19 vaccination equity index measure, calculated as the relative ratio of vaccinations to deaths by zip code in Chicago through July 2022. The map outlines in bold the 7 zip codes that experienced improvements of more than 0.1 as measured by this equity index from June 2021 to July 2022. Sources: Chicago Department of Health and US Census Bureau.

## Background

As of July 2022, a year and a half into the COVID-19 vaccination campaign, about 70% of Chicago, Illinois, residents have completed a primary vaccination series, and nearly 8,000 Chicagoans have died from COVID-19 (1). Stark disparities exist in the burden experienced across Chicago; higher case and mortality rates are found in areas with higher social vulnerability (2), mostly Black or Hispanic residents (3–5), and residents with lower educational attainment (6). Stark differences also exist in vaccination rates, with higher rates in higher-income, largely White communities (7).

We combined vaccination and death data to visualize geographic patterns for an equity index, calculated as the relative ratio of cumulative vaccinations to deaths by zip code. This metric is based on the principle that populations experiencing disproportionate burdens should be prioritized for risk-reducing interventions and is based on earlier work on HIV prevention (8) and community-based COVID-19 vaccine equity (9). Our prior work in support of The Rockefeller Foundation’s Equity-First Vaccination Initiative used this metric to analyze COVID-19 vaccination equity by race and ethnicity in Chicago and 4 other cities (10,11).

In this article, we focus on geographic equity within Chicago. Our maps, paired with socioeconomic data, aim to identify geographic patterns and characteristics of zip codes with higher and lower equity index values, as well as those that have marked the most progress toward equity over time.

## Data and Methods

We downloaded data on COVID-19 vaccinations and deaths by zip code from the Chicago Department of Public Health (12). We summed counts of people completing a primary vaccination series and deaths due to COVID-19 at 2 points: the end of June 2021, when most people aged 12 years or older were eligible to complete their primary series, and the end of July 2022.

We constructed an equity index to assess whether vaccination uptake was proportional to deaths from COVID-19. We calculated this metric for each zip code, with a numerator of the zip code’s share of people citywide who completed a primary series, cumulative through June 2021 and July 2022, and a denominator of the zip code’s share of deaths due to COVID-19 through those same dates. For example, if a zip code accounted for 5% of both citywide deaths and people completing a primary series, its equity index would be 1.

Zip codes experiencing a disproportionate share of deaths relative to vaccinations have index values below 1; for example, a zip code with 4% of vaccinated people and 5% of deaths would have an equity index value of 0.80. Zip codes accounting for a larger share of the vaccinated population than deaths have index values greater than 1; for example, a zip code with 5% of vaccinated people and 4% of deaths would have an equity index value of 1.25. (By construction, the equity index for Chicago overall is always 1.) Convergence of zip code equity index values toward 1 over time would indicate movement toward geographic equity in Chicago.

To highlight segregation and its relationship with the index, we mapped equity index values as of July 2022 using R (R Foundation) across 4 panels corresponding to the racial or ethnic majority of each zip code. The 2 bottom panels of the map together account for the full set of zip codes in our analyses. We outlined the 7 zip codes with improvements of more than 0.1 since June 2021. We analyzed characteristics of zip codes by groupings of equity index values using American Community Survey data, including race or ethnicity distribution, educational attainment, median household income, and share with health insurance (13). Prior research suggests that these characteristics relate to COVID-19 mortality and vaccination rates (3–7); moreover, most are among the factors used to calculate Social Vulnerability Index values and therefore are recognized as relevant to understanding geographic inequities (14). Consistent with prior work (7), we excluded 6 zip codes mostly outside Chicago or with populations with fewer than 10,000 people.

## Highlights

The map shows stark and persistent inequities when accounting for both vaccination rates and disease burden across Chicago and highlights the spatial concentration of zip codes with disproportionate deaths relative to their shares of the vaccinated population. Of the 52 zip codes in the analysis, 15 include mostly Black residents. Most of these 15 majority Black zip codes had equity index values below 0.7, and only 2 (near the University of Chicago) had index values greater than 1. Chicago’s 7 majority Hispanic zip codes had equity index values just below or just above 1. Conversely, of the 17 zip codes with index values greater than 1.2, all but 1 were majority White or Asian.

Zip codes with equity index increases of more than 0.1 since June 2021 are marked with a thick black outline. Chicago’s majority Hispanic zip codes account for 4 of these 7 zip codes. Only 2 zip codes that include mostly Black residents experienced increases of more than 0.1.

The 8 zip codes with index values below 0.7 are nearly 90% Black and have the lowest median household incomes, although their percentage of residents with health insurance and postsecondary education are higher than zip code groupings with index values near 1 (Table 1). The 17 zip codes with index values greater than 1.2 have median household incomes approaching $100,000 per year, about two-thirds of residents have education beyond high school, and 95% have health insurance. The 6 zip codes with index values below 1 in June 2021 but increases of more than 0.1 were on average about two-thirds Hispanic and had higher percentages of residents without health insurance and without post–high school education than any other group (Table 2).

**Table 1:** Demographic and Socioeconomic Characteristics of Zip Codes^a^ in Chicago, Grouped by COVID-19 Vaccination Equity Index, July 2022

Demographic/socioeconomic characteristic	Vaccination Equity Index^b^				
	<0.70	0.70–0.79	0.80–0.99	1.00–1.25	>1.25
No. of ZIP codes	8	7	11	9	17
Population, no.	393,491	368,893	532,752	636,386	744,037
Total population, %	14.7	13.8	19.9	23.8	27.8
Cumulative vaccinated through July 2022, %	11.9	13.0	20.0	25.1	29.9
Cumulative deaths through July 2022, %	22.8	17.4	22.2	23.2	14.4
Race and ethnicity, %					
Asian	0.3	2.6	5.1	11.4	9.4
Black	89.1	42.0	18.2	16.1	8.7
Hispanic	4.3	35.7	40.0	43.4	17.2
White	4.5	18.0	34.7	26.6	61.1
Average median household income, $	40,400	43,600	63,711	55,062	93,316
Postsecondary education^c^, %	51.6	44.2	48.8	45.4	66.4
Health insurance, %	91.3	88.2	88.8	87.8	94.5

a Values for groupings of zip codes reflect population-weighted averages of zip code-level data. We excluded 6 zip codes at least partially in the City of Chicago but that either lie mostly outside of the city or have fewer than 10,000 people. Sources: Chicago Department of Health (12); US. Census Bureau, American Community Survey, 2016–2020, 5-year estimates (13).

b Equity index measure calculated as the relative ratio of vaccinations to deaths by zip code in Chicago. For example, a zip code with above-average death rates and below-average vaccination rates would have an equity index below 1. By contrast, a zip code with above-average vaccination rates and below average death rates will have an equity index above 1.

c Share with postsecondary education refers to people aged 25 years or older.

**Table 2:** Change in Vaccination Equity Index of Zip Codes^a^ in Chicago From June 2021 to July 2022, by Vaccination Equity Index^b^

Demographic/socioeconomic characteristic	Change in index for zip codes with equity index values <1			Change in index for zip codes with June 2021 equity index values ≥1		
	< −0.1	−0.1 to 0.1	>0.1	< −0.1	−0.1 to 0.1	>0.1
No. of zip codes	0	23	6	14	8	1
Population	—	1,140,099	459,625	508,983	552,339	14,513
Total population, %)	—	42.6	17.2	19.0	20.6	0.5
Cumulative vaccinated, through June 2021, %	—	37.5	16.1	22.9	22.8	0.8
Cumulative deaths, through June 2021, %	—	52.6	21.8	8.2	17.2	0.2
Cumulative vaccinated, through July 2022, %	—	39.6	18.1	20.1	21.6	0.7
Cumulative deaths, through July 2022, %	—	54.7	19.9	8.5	16.6	0.2
Race and ethnicity, %						
Asian	—	5.0	1.8	10.7	10.4	24.3
Black	—	44.5	25.8	11.5	15.2	4.7
Hispanic	—	21.4	64.9	9.8	31.2	8.6
White	—	26.7	6.5	64.5	40.1	59.4
Average median household income, $	—	54,928	42,540	93,786	70,413	109,625
Postsecondary education^c^, %	—	51.7	33.5	69.0	55.0	68.0
Health insurance, %	—	90.2	85.0	95.7	90.5	97.0

Abbreviation: — , not applicable.

a Values for groupings of zip codes reflect population-weighted averages of zip code–level data. We excluded 6 zip codes at least partially in the City of Chicago but that either lie mostly outside of the city or have fewer than 10,000 people. Sources: Chicago Department of Health (12); US Census Bureau, American Community Survey, 2016–2020, 5-year estimates (13).

b Equity index measure calculated as the relative ratio of vaccinations to deaths by zip code in Chicago. For example, a zip code with above-average death rates and below-average vaccination rates would have an equity index below 1. By contrast, a zip code with above-average vaccination rates and below average death rates will have an equity index above 1.

c Share with postsecondary education refers to people aged 25 years or older.

No zip codes with index values below 1 as of June 2021 experienced substantial decreases (of more than 0.1) over the next 13 months. Of the 26 zip codes with index values at or above 1 as of June 2021, 14 had decreases of that magnitude, but only 1 dropped below 1 (going from 1.07 to 0.92). By necessity, as zip codes with index values below 1 in June 2021 made progress toward equity (by increasing), index values in zip codes that initially were above 1 had to decrease. Both are indicative of movement toward citywide equity (Table 2).

## Action

Prior studies report higher case and mortality rates (3–6) and lower rates of vaccination in the early stages of the vaccination campaign (7) among Black and Hispanic populations in Chicago. We found that as of July 2022 Chicago neighborhoods with mostly Black residents were experiencing persistent inequities when accounting for COVID-19 vaccinations and deaths. Vaccine supply, vaccination demand, and the effects of lower vaccination rates on subsequent death rates contribute to this finding, and future research could explore which factors are most relevant. Despite this, we identified several majority Hispanic or Black zip codes that experienced equity index improvements since 2021. We noted that the zip codes with the largest improvements were mostly Hispanic and had both lower educational attainment and shares with health insurance than zip codes that saw little change over time; explanations for these observed patterns merit further investigation as well.

Our analyses of zip codes based on their racial or ethnic makeup obscures variation within racial and ethnic groups, and a more thorough analysis of differences within Hispanic and Asian populations may inform more tailored public health interventions. As the vaccination campaign transitions to an ongoing effort to encourage booster uptake, public health officials should explore whether there are promising practices that yielded successes in some neighborhoods and apply those to communities where inequities remain deeply entrenched. Our prior work in support of the Equity-First Vaccination Initiative suggests several such promising practices, including investigating barriers to vaccination, partnering with trusted messengers in a community, and co-creating messaging and information campaigns with members of the community (10). Additionally, as the use of real-time data dashboards to communicate information and target responses expands (15), officials might consider incorporating an equity index measure into these data resources.
